# Wnt Signaling Pathway Collapse upon β-Catenin Destruction by a Novel Antimicrobial Peptide SKACP003: Unveiling the Molecular Mechanism and Genetic Activities Using Breast Cancer Cell Lines

**DOI:** 10.3390/molecules28030930

**Published:** 2023-01-17

**Authors:** Kanitha Selvarathinam, Prabhu Subramani, Malarvili Thekkumalai, Ravikumar Vilwanathan, Ramganesh Selvarajan, Akebe Luther King Abia

**Affiliations:** 1 Department of Biochemistry, J.J. College of Arts and Science (Autonomous), Pudukkottai 622422, Tamilnadu, India; 2 Department of Biochemistry, School of Life Science, Bharathidasan University, Tiruchirappalli 622422, Tamilnadu, India; 3 Government Arts and Science College, Tiruchirappalli 622422, Tamilnadu, India; 4 Department of Environmental Sciences, College of Agricultural and Environmental Sciences (CAES), University of South Africa (UNISA), Florida—Campus, Florida Park, Roodepoort 1709, South Africa; 5 Laboratory of Extraterrestrial Ocean Systems (LEOS), Institute of Deep-Sea Science and Engineering, Chinese Academy of Sciences (CAS), Sanya 572000, China; 6 Environmental Research Foundation, Westville 3630, South Africa

**Keywords:** antimicrobial peptides, anticancer agents, breast cancer, Wnt pathway, β-catenin, downregulation, oncology, alternative therapy

## Abstract

Despite progress in breast cancer treatment, the survival rate for patients with metastatic breast cancer remains low due to chemotherapeutic agent resistance and the lack of specificity of the current generation of cancer drugs. Our previous findings indicated that the antimicrobial peptide SKACP003 exhibited anticancer properties, particularly against the MCF-7, MDA-MB-231, and MDA-MB-453 breast cancer cell lines. However, the mechanism of SKACP003-induced cancer cell death is unknown. Here, we investigated the molecular mechanism by which SKACP003 inhibits the cell cycle, cell proliferation, and angiogenesis in breast cancer cell lines. The results revealed that all the breast cancer cell lines treated at their IC_50_ values significantly inhibited the replicative phase of the cell cycle. The SKACP003-induced growth inhibition induced apoptosis, as evidenced by a decrease in BCL-2 and an increase in BAX and caspase gene (Cas-3, Cas-8, and Cas-9) expression. Reduced expression of the β-Catenin signaling pathway was associated with the SKACP003-induced apoptosis. SKACP003-treated breast cancer cells showed decreased expression of Wnt/β-Catenin targeting genes such as C-Myc, P^68^, and COX-2 and significant downregulation of CDK-4 and CDK-6 genes. Furthermore, cytoplasmic β-catenin protein levels in SKACP003-treated cell lines were significantly lower than in control cell lines. The results of the current study suggest that the newly identified antimicrobial peptide SKACP003 has great potential as a candidate for specifically targeting the β-catenin and thus significantly reducing the progression and prognosis of breast cancer cell lines.

## 1. Introduction

The prevalence of breast cancer among women results in a significant reduction in their average lifespan. Similarly, the mortality ratio is skewed in favor of urban women compared to their rural counterparts. Most concerning is that, as per estimates, the number of women impacted by this problem is expected to rise exponentially in the near future [1]. Regarding treatment, a systematic approach is employed to treat the ailment effectively. Chemotherapy is currently one of the most widely used treatment methods. However, it has some drawbacks, such as ineffectiveness against cancerous cells due to a lack of target specificity, which destroys adjoining healthy cells and has adverse side effects [2,3]. Conversely, immunotherapy, a promising alternative for treating cancer, has some negative side effects like toxicity and reverse autoimmunity and rarely reaches its intended target tissues [4]. In addition to these limitations, modern cancer therapy has difficulties coping with cancer cells that have developed multidrug resistance after metastasizing [5].

Consequently, it is imperative to investigate alternative therapies involving an oncolytic agent as a potential targeted therapy against cancer cells. Antimicrobial peptides (AMPs) are a promising option in this regard (a more meaningful option) because AMPs have been shown to provide the necessary immunity against a wide variety of pathogens [6]. More importantly, these antimicrobial peptides are naturally produced by bacteria, fungi, plants, and animals such as arthropods, fish, amphibians, and mammals. However, only a few of these AMPs are found to be cationic and hydrophobic in their natural state [7,8]. Nevertheless, recent studies on the anticancer activities, selectivity, and efficacy of AMPs, especially in preclinical studies, greatly support the idea of AMPs as practical, novel oncogenic agents for cancer therapy [9,10,11,12,13].

Anticancer peptides (ACPs) are short antimicrobial peptides (5–40 amino acids) with anticancer properties. ACPs generally have a low molecular weight and a cationic nature at physiologically relevant pH levels. These ACPs have a high degree of precision in their action and can easily penetrate tumor walls. Furthermore, they are easily synthesized in the laboratory in large quantities. With the characteristics mentioned earlier and the ability to interact only with cancerous cells while leaving healthy cells, ACPs can be considered a critical component in cancer treatment [8,14]. By taking advantage of the abundance of ACPs possessing anticancer properties, it is possible to design new drugs for targeted therapy. In recent years, a limited number of ACPs have been granted clinical approval for cancer treatment [15], prompting researchers to explore more peptides with enhanced performance.

Although several signaling pathways are available in the literature to explain the progression of breast cancer, the Wnt signaling pathway is the most comprehensive. The Wnt family of glycoproteins determines cell fate during embryonic development and maintains tissue homeostasis in adult organisms [16]. Without the Wnt ligand, glycogen synthase kinase-β (GSKβ) and casein kinase 1 phosphorylate β-catenin, accelerating its degradation via the ubiquitination pathway [17]. On the other hand, the presence of the Wnt ligand prevents β-catenin phosphorylation, thereby impeding its degradation. Consequently, there is an accumulation of unphosphorylated β-catenin within the cytoplasm. This results in the transfusion of β-catenin into the nucleus, which affects mRNA synthesis for many Wnt-regulated genes, including cyclin D1 and C-Myc, and thus initiates tumorigenesis [18,19]. Studies have demonstrated that the mouse mammary tumorigenesis model showed an active Wnt signaling cascade [20,21]. Therefore, the most effective breast cancer prevention and treatment method may involve interrupting the sequence. More specifically, the Wnt signaling pathway and oncogenesis can be disrupted by blocking β-catenin accumulation in the cytoplasm. It is worth noting that Wnt signaling is also a driving force in the development of many other cancers. Presumably, disrupting the essential activities of β-catenin in the Wnt signaling pathway [22,23] can result in the desired elimination of cancerous cells, and thus β-catenin can be viewed as a crucial target in the field of breast cancer therapy [23,24,25,26,27,28,29].

This study builds on our previous work [30], which focused on targeting β-catenin, a key component of the Wnt signaling pathway, with the novel anticancer peptide SKACP003. In that study, we found that SKACP003 induced dose-dependent cytotoxicity in human breast cancer cell lines, with increased DNA damage seen across the cell lines studied. The evaluation of the molecular level, however, was not investigated. This study aimed to probe the molecular mechanism by which SKACP003 inhibits the growth of MCF-7, MDA-MB-231, and MDA-MB-453 breast cancer cell lines. The pathway promoting the breakdown of cancer activity was measured by assessing the inhibition activity of SKACP003 towards the β-catenin protein by measuring the downregulation of its downstream target genes.

## 2. Results

### 2.1. Cell Cycle Arrest by Synthesized Peptide

**Figure 1:**
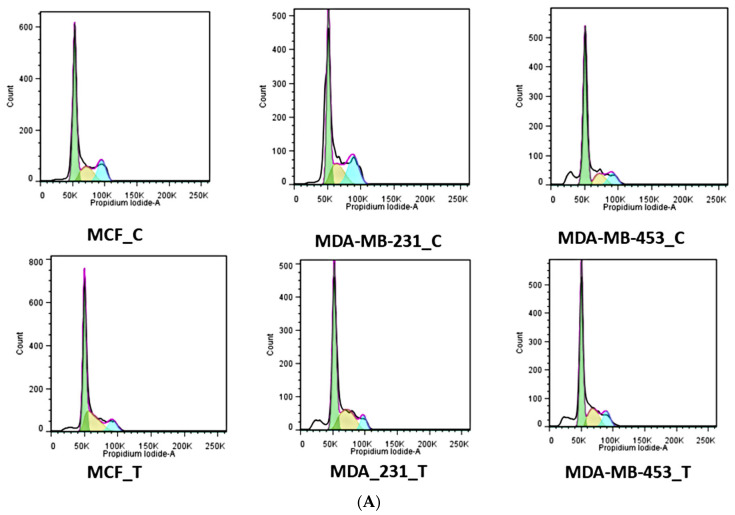

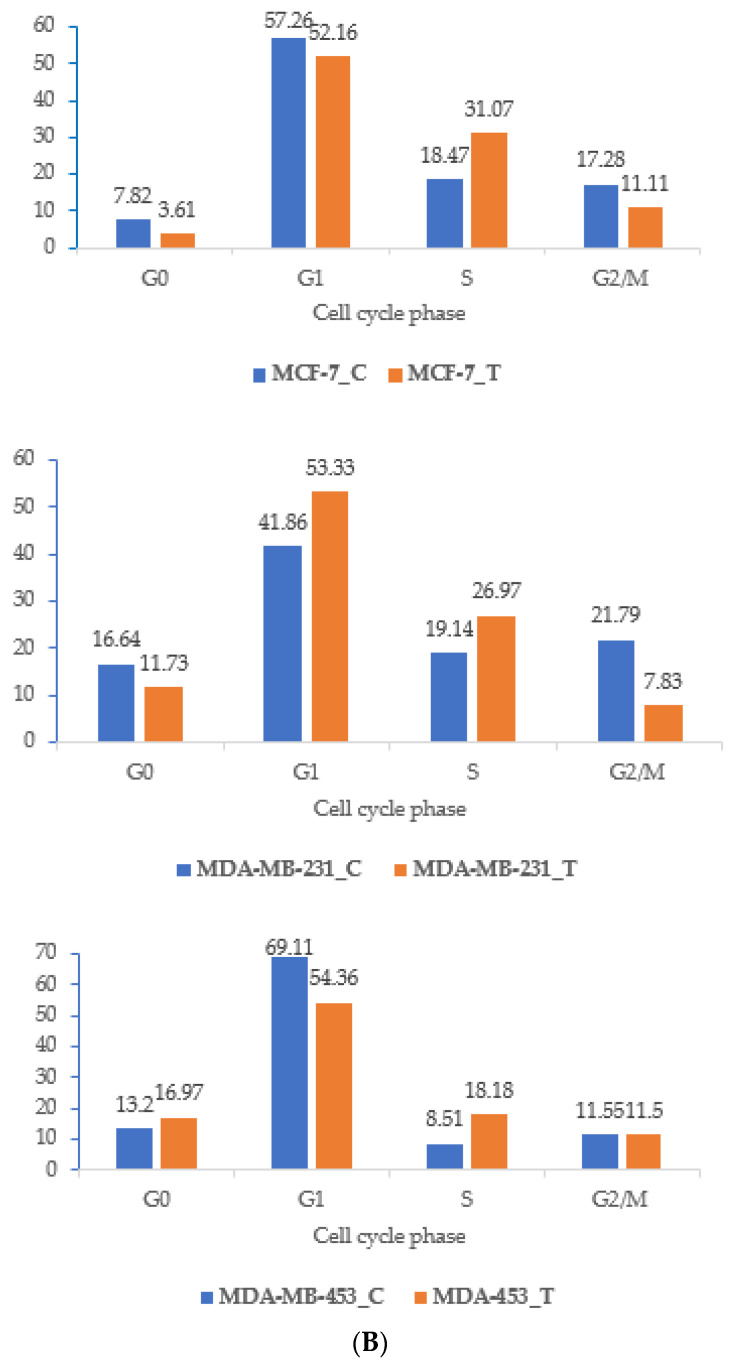
Cell cycle analysis (**A**) The cell cycle of the breast cancer cell lines MCF-7, MDA-MB-231, and MDA-MB-453 were analyzed using flow cytometry on the control and the SKACP003treated cell lines. (**B**) The ratio of cells in the G0, G1, S, and G2/M cell cycle phases in the control and the SKACP003-treated MCF-7, MDA-MB-231, and MDA-MB-453 cell lines.

Cancer cells arrested in distinct cell cycle phases such as G1, S, and G2 were studied using flow cytometry. Cells treated with SKACP003 were compared to untreated controls for their accumulation in distinct cell cycle stages. Each of the three breast cancer cell lines studied showed an increase in the proportion of cells in the synthetic phase (S phase) after treatment with the peptide SKACP003 at its IC_50_ concentration (Figure 1A). In addition, SKACP003 treatment resulted in a greater percentage of cells in the G0-G1 cell cycle phase than controls (Figure 1B), most notably in the breast cancer cell line MDA-MB-453. The results showed that a significant number of cells accumulated in the S phase after treatment with SKACP003 in several breast cancer cell lines, which is thought to play a critical role in cell cycle regulation (Figure 1). This mode of cell cycle arrest is promoted by the DNA fragmentation assay.

### 2.2. Down-Regulation of CDK-4 and CDK-6 Expressions by SKACP003 in Breast Cancer Cell Lines

**Figure 2:**
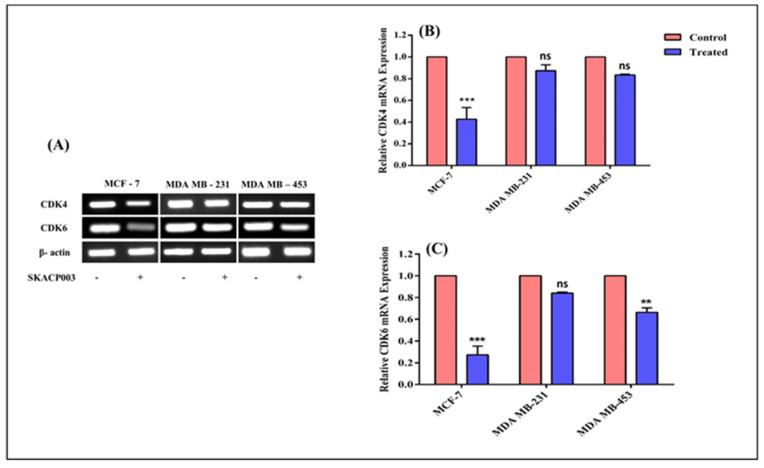
Down-regulation of CDK-4 and CDK-6 expressions by SKACP003 in breast cancer cell lines. The mRNA levels of CDK-4 and CDK-6 from total RNA were analyzed by RT-PCR for the control and SKACP003-treated breast cancer cell lines of MCF-7, MDA-MB-231, and MDA-MB-453. (**A**) RT-PCR products; (**B**) downregulation of CDK-4 by SKACP003; (**C**) downregulation of CDK-6 by SKACP003. ** = statistical significance at *p* < 0.01; *** = statistical significance at *p* < 0.001; ns = not significant.

The expression levels of CDK-4 and CDK-6 increase to a greater extent in violent malignancy triggered by the Wnt/β-catenin signaling pathway. Therefore, to determine the precise molecular mechanism involved in inhibiting the proliferation of breast cancer cell lines, MCF-7, MDA-MB-231, and MDA-MB-453 were treated with SKACP003 at their respective IC_50_ concentrations for 24 h, and the corresponding expression of CDK-4 and CDK-6 genes was evaluated. After treatment with SKACP003, the CDK-4 and CDK-6 genes were significantly downregulated in the breast cancer cell lines compared to the control cell lines (Figure 2), indicating an anti-proliferative activity in all breast cancer cell lines tested.

### 2.3. Down-Regulation of C-Myc, P^68^ and COX-2genes Expression by SKACP003

The β-catenin/TCF-4 complex regulates the P^68^ promoter. The expression of the P^68^ gene in the Wnt signaling pathway involves the role of C-Myc-and COX-2, which, in turn, is regulated by the β-catenin protein [31]. β-catenin stabilization and subsequent nuclear accumulation through Wnt signaling are vital for the upregulation of the C-Myc, P^68^, and COX-2 gene expression, a major contributing factor in breast cancer progression. Apart from the transcriptional activation of the target gene expression by the β-catenin, the stabilization of unstable mRNAs, like COX-2, may raise the expression of target mRNAs in malignant cells.

**Figure 3:**
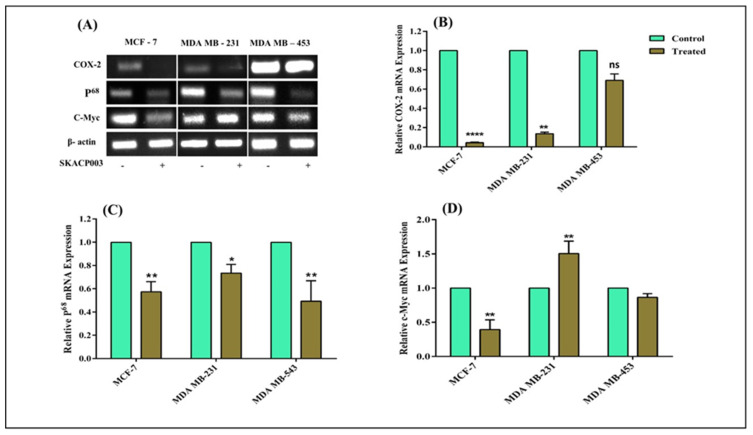
Downregulation of expressions of (**B**) COX-2, (**C**) P^68^, and (**D**) C-Myc genes by SKACP003. The mRNA levels of COX-2, P^68^, and C-Myc from total RNA were analyzed by RT-PCR for the control and SKACP003-treated breast cancer cell lines of MCF-7, MDA-MB-231, and MDA-MB-453 (**A**). * = statistical significance at *p* < 0.05; ** = statistical significance at *p* < 0.01; **** = statistical significance at *p* < 0.0001; ns = not significant.

Thus, it becomes necessary to analyze the impact of the reduction or destruction of β-catenin by SKACP003 in all three breast cancer cell lines to confirm the downregulation of the three genes mentioned above. Therefore, the influence of SKACP003 on C-Myc, P^68^ and COX-2 was investigated, and the downstream target genes of β-catenin protein, which play a significant part in the progression of breast carcinoma. The tested peptide curtailed the expression of the C-Myc, P^68^, and COX-2 genes extensively in all cell lines except MDA-MB-231, where C-Myc was up-regulated (Figure 3), thereby retarding breast cancer development and progression.

### 2.4. SKACP003 Reduces Cell Migration

Wnt/β-catenin pathway modulates the expression of the proteins like matrix metalloproteinase (MMP-2 and MMP-7) and vascular endothelial growth factor (VEGF-A) that promote metastasis and angiogenesis [32]. Previous research has shown the Wnt/β-catenin signaling pathway directly controls the MMP promoter via LEF/TCF binding. MMP-2, MMP-7, and VEGF-A are the major genes that promote metastasis and angiogenesis in breast cancer cells.

**Figure 4:**
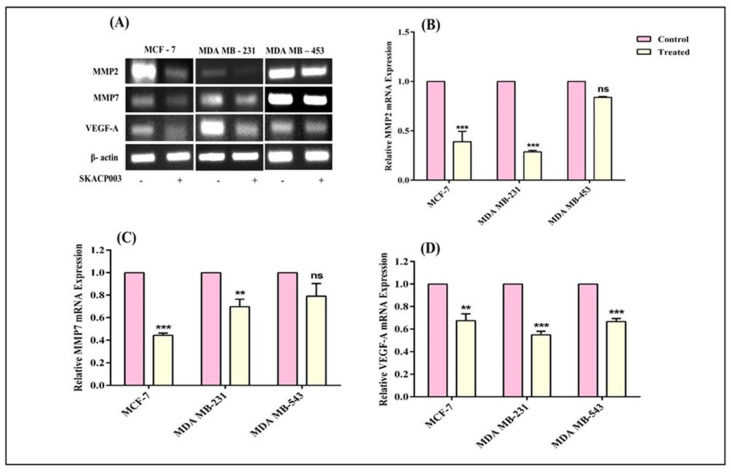
Inhibition of metastasis and angiogenesis by SKACP003. The mRNA levels of MMP-2 (**B**), MMP-7 (**C**), and VEGF-A (**D**) from total RNA were analyzed by RT-PCR (**A**) for the control and SKACP003-treated breast cancer cell lines of MCF-7, MDA-MB-231, and MDA-MB-453. ** = statistical significance at *p* < 0.01; *** = statistical significance at *p* < 0.001; ns = not significant.

Therefore, the current study employed RT-PCR analysis to assess MMP-2, MMP-4, and VEGF-A gene expression in response to SKACP003 and confirmed that treatment significantly decreased the expression of all MMP genes and the VEGF-A gene. The results also showed that the SKACP003 successfully inhibited cell migration in all tested breast cancer cell lines (Figure 4).

### 2.5. SKACP003 Induced Apoptosis in MCF-7, MDA-MB-231 and MDA-MB-453 Breast Cancer Cell Lines

**Figure 5:**
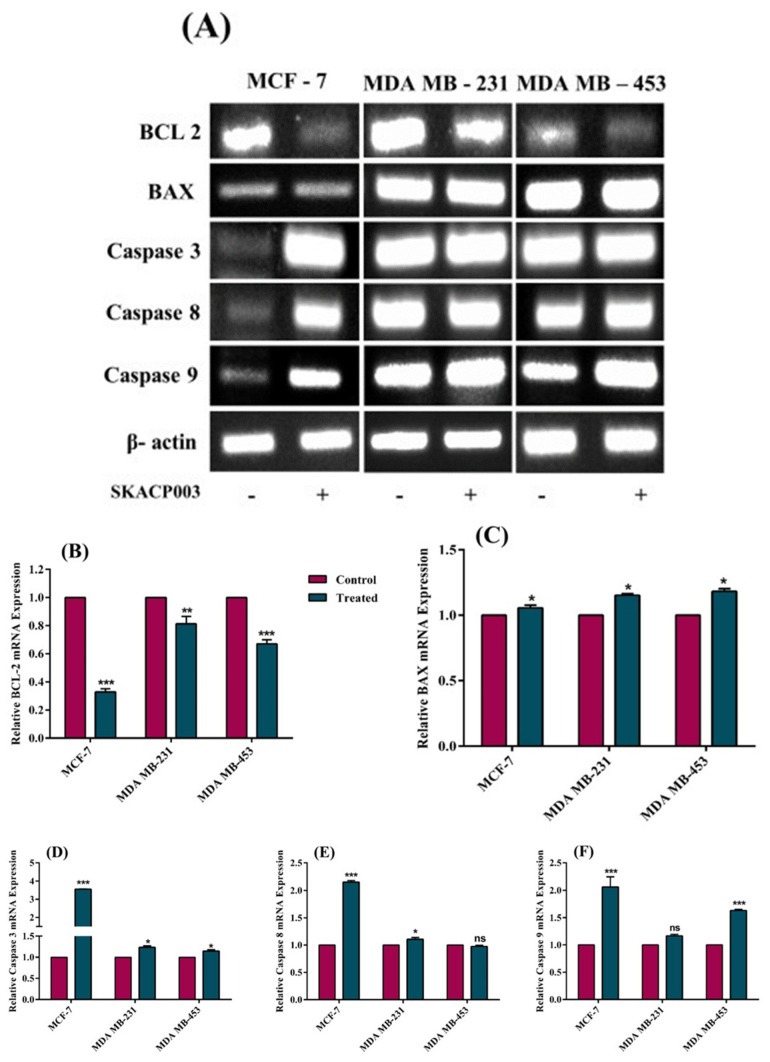
Effect of SKACP003 on the apoptotic pathway of the breast cancer cell lines. RT-PCR (**A**) was used to analyse the mRNA levels of (**B**) BCL-2, (**C**) BAX, (**D**) Cas-3, (**E**) Cas-8, and (**F**) Cas-9 from total RNA for the control and SKACP003-treated breast cancer cell lines of MCF-7, MDA-MB-231, and MDA-MB-453. * = statistical significance at *p* < 0.05; ** = statistical significance at *p* < 0.01; *** = statistical significance at *p* < 0.001; ns = not significant.

In general, apoptosis is inhibited or downregulated in almost all cancer cells. So, the efficacy of SKACP003 as a vital anticancer agent is determined in terms of its ability to induce cancer cell death in the treated cell lines. The expression of BCL-2, BAX, Cas-3, Cas-8, and Cas-9 genes is useful for studying apoptosis because these genes are thought to be the most important players in the process. Consequently, the effects of SKACP003 on the expression of the genes mentioned above were examined. As seen in Figure 5, apoptosis resulted in all the treated breast cancer cell lines, as evidenced by significant decreases in BCL-2 levels and increases in BAX levels. However, the unaltered Cas-8 and Cas-9 expression levels in cell lines MDA-MB-231 and MDA-MB-543, respectively, indicate that SKACP003 followed a path that did not necessitate caspase. Incidentally, it is known that caspase-dependent cell death is not the only pathway to induce apoptosis. Therefore, the present study points out the intriguing possibility of cancer cell growth arrest leading to caspase-independent cell death.

### 2.6. Effect of SKACP003 on the Cytoplasmic β-Catenin Level

**Figure 6:**
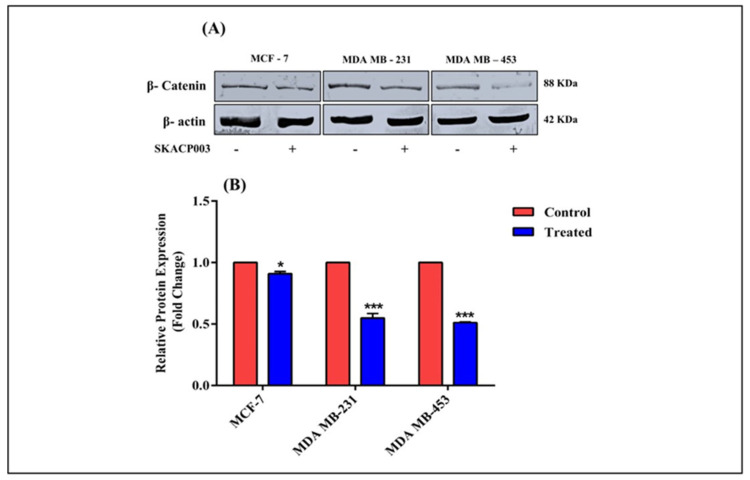
(**A**) Western blot analysis of β-catenin and control protein. (**B**) A bar chart displaying the level of β-catenin protein control and treated breast cancer cell lines. * = statistical significance at *p* < 0.05; *** = statistical significance at *p* < 0.001.

The direct inhibitory effect of SKACP003 on the cytoplasmic β-catenin level has been shown in our previous studies using in silico and experimental evaluations such as the MTT (3-[4,5-dimethylthiazol-2-yl]-2,5 diphenyl tetrazolium bromide) assay, staining procedures, cell cycle assay, and the expression of the downstream genes that are regulated by β-catenin protein [30]. As a result, the amount of cytoplasmic β-catenin protein in SKACP003-treated breast cancer cell lines was examined to provide further confirmation of our previous results. As a result, the cytoplasmic β-catenin protein level was significantly reduced in SKACP003-treated cell lines compared to control cell lines (Figure 6).

## 3. Discussion

Wnt signaling is vital because it promotes tumor progression by upregulating various critical factors. Furthermore, the deregulation of Wnt signaling is the primary indicator of most cancers, including breast cancer. To date, even a modest amount of knowledge about the Wnt signaling pathway’s function in cancer has contributed to the advancement of effective therapeutic alternatives [33,34,35,36,37], although only a few have advanced to early clinical trials. The most pressing requirement is for the drug to selectively attack the Wnt signaling pathway in cancer cells while avoiding collateral damage to healthy cells [34,35,36]. As a result, researchers are establishing novel strategies to target the Wnt molecules specifically expressed by cancer cells. Among the various components of Wnt signaling, β-catenin plays a key role in tumorigenesis. Several studies into the role of β-catenin protein accumulation and its translocation into the nucleus in the initiation and development of breast cancer have yielded important insights [22,26,27,38,39].

Recent studies have shown that AMPs can selectively target and kill cancer cells while leaving healthy cells [40,41,42]. Several types of cancer, including those of the lung [43], cervix [44], liver [45], prostate [46], and breast [47], are currently being studied in conjunction with AMPs. AMPs have an effect by increasing cell death via apoptosis or necrosis. However, few AMPs have been found to damage the intracellular components of malignant cells. Additionally, some peptides kill cancer cells due to their complexity via multiple mechanisms [30].

As a follow-up to our previous work on identifying the novel antimicrobial peptide, SKACP003, which targeted the β-catenin and curtailed the Wnt signaling pathway, the present study attempted to understand better the inhibitory action of SKACP003 against β-catenin protein and its downstream target genes.

### 3.1. Cell Cycle Arrest by Synthesized Peptide

The current study used three human breast cancer cell lines: MCF-7, MDA-MB-231, and MDA-MB-453. Cell lines treated with the peptide analogue showed significant inhibition of DNA synthesis after 24 h, followed by significant inhibition of cell growth after 48 h. Only viable cells were counted during the growth studies [48], indicating that cell growth inhibition appears to be cytostatic rather than cytotoxic. Loss of cell-cycle checkpoint controls, which normally regulate cell division, has been linked to cancer progression. These checkpoints ensure DNA integrity and the coordinated expression of genes [49]. In the current investigation, treatment with the SKACP003 analogue resulted in a statistically significant accumulation of cells in the G0/G1 phase across all cell lines tested (Figure 1). Control and SKACP003-treated cells were compared for their relative accumulation of cells in distinct phases of the cell cycle. A higher proportion of cells in the S phase was seen after treatment with IC 50 concentrations of SKACP003 across all three breast cancer cell lines, compared to the control. It was also noted that the percentage of cells in the G2/M phase was lower in the treated cells than in the control group. This demonstrates that SKACP003 induces cell cycle arrest in the S phase, which is linked to apoptosis. SKACP003 treatment of the breast cancer cell lines clearly increased the proportion of cells in the S phase, an essential activity in cell cycle regulation. This pattern of cell cycle arrest is strongly associated with DNA fragmentation, as evidenced by a DNA fragmentation assay. Thus, this assay provides compelling evidence that SKACP003 can successfully arrest the breast cancer cell lines in their S phase, warranting further investigation. By supplementing the existing cell-cycle machinery with extrinsic cell-cycle regulators, such as SKACP003 AMPs, it may be possible to prevent the initiation or progression of cancer growth.

### 3.2. Down-Regulation of CDK-4 and CDK-6 Expressions by SKACP003 in Breast Cancer Cell Lines

Cell cycle analysis reveals implications that necessitate further investigation into the molecular mechanism responsible for cell cycle arrest. Deregulated activation of cell division kinases (CDKs) has been linked to erratic cell division, prompting the present study to examine the expression of CDK-4 and CDK-6 genes. According to the results, SKACP003 was able to suppress the expression of CDK-4 and CDK-6 in breast cancer cell lines (Figure 2). Increased β-catenin activity alone causes overexpression of CDK-4 and CDK-6 genes [38,39,50], indicating that SKACP003 deactivates β-catenin. 

### 3.3. Down-Regulation of C-Myc, P^68^ and COX-2genes Expression by SKACP003

Studies on the expression of oncogenes, such as C-Myc, P^68^, and COX-2, suggested that they were significant factors in breast cancer progression [51,52]. C-Myc plays a role by acting as a transcription factor [53,54,55,56]. All treated breast cancer cell lines showed decreased cancerous activity. Reduced expression of the genes above, whose expression was crucially regulated by the β-catenin protein, suggests that β-catenin was deactivated by SKACP003, which may be considered a useful tool for halting the development and progression of breast cancer. In addition, a notable decrease in COX-2 gene expression (Figure 3) suggests that SKACP003 targeted β-catenin and abolished the stabilization of unstable COX-2 mRNA function by the same [57]. Based on these findings, SKACP003 may be a promising candidate for cancer treatment because it effectively detuned all β-catenin-dependent oncogenic activities.

### 3.4. SKACP003 Reduces Cell Migration

Through the action of β-catenin, an additional significant process that must be taken into account regarding the Wnt signaling pathway is the stimulation of the expression of proteins that play a role in the angiogenesis process, such as MMP and VEGF-A [58]. Therefore, the study investigated whether the inhibition of β-catenin protein favored a decrease in the expression of crucial angiogenesis-promoting factors, such as VEGF-A and MMP, in all treated breast cancer cell lines. All the treated breast cancer cell lines demonstrated a substantial reduction in MMP-2, MMP-7, and VEGF-A gene expression levels (Figure 4). Considering that affected individuals acquire chemoresistance due to the over-expression of VEGF-A via the Wnt signaling pathway, the effect of SKACP003 on VEGF-A under-expression has become more significant.

### 3.5. SKACP003 Induced Apoptosis in MCF-7, MDA-MB-231 and MDA-MB-453 Breast Cancer Cell Lines

Developing an effective anticancer drug must consider apoptosis, or programmed cell death. Previous research from our research group demonstrated that SKACP003 induced apoptosis in all breast cancer cell lines [30]. However, the apoptosis mechanism was not considered in the meantime. To determine the underlying molecular mechanism of cell death, we analyzed how treatment of breast cancer cell lines affected the expression of genes involved in apoptosis, such as BCL-2, BAX, Cas-3, Cas-8, and Cas-9. In general, cell death can be caspase-dependent or caspase-independent. The current results show that SKACP003 could degrade β-catenin in breast cancer cell lines without affecting cas-8 and cas-9 expressions. Specifically, it was discovered that the BCL-2 expression level declined while that of BAX increased. In addition, cas-3 expression was up-regulated in treated MDA-MB-231 and MDA-MB-453 breast cancer cell lines but not in MCF-7 (Figure 5). This is consistent with previous findings [59] that MCF-7 did not express CASP-3. While BCL-2 and BAX are members of the same family of proteins, BCL-2 inhibits apoptosis while BAX promotes it. However, both are crucial regulators of mitochondrial functions, including membrane permeability and the export of cytochrome-C from mitochondria to the cytosol [60]. Under normal conditions, BCL-2 is mostly found in the nucleus, mitochondria, and endoplasmic reticulum, whereas BAX is mostly found in the cytoplasm [61]. Upon stimulation, BAX translocates into the mitochondria, decreasing the membrane potential and increasing permeability. This initiates a mitochondrial caspase-independent apoptosis pathway by releasing apoptotic entities from the mitochondrial intermembrane space into the cytosol, which are then transported into the nucleus, where they bind to the DNA, causing nuclear condensation and DNA fragmentation [62]. All the above findings and findings from our earlier staining procedures [30] provide sufficient evidence to conclude that SKACP003 induces only a caspase-independent cell death process [13,63].

### 3.6. Effect of SKACP003 on the Cytoplasmic β-Catenin Level

The Western blot study adds to the evidence that SKACP003 inhibits cancer cells growth by selectively attacking and destroying the cytoplasmic β-catenin protein (Figure 6). Overall, SKACP003 hinders the development and prognosis of various types of breast cancer, showing optimistic hope of finding solutions for the most aggressive triple-negative type of breast cancer.

## 4. Materials and Methods

All the cell culture supplies, including Dulbecco’s modified Eagle’s medium (DMEM), 10% fetal bovine serum (FBS), penicillin/streptomycin, phosphate-buffered saline (PBS), and dimethyl sulfoxide (DMSO), were purchased from Hi-Media Laboratories, India. The cDNA Synthesis Kit, TRIzol reagent, and Emerald PCR Master Mix were acquired from Takara, Japan. The β-catenin antibody (SC-376841), produced from mice, and β–actin antibody (SC-8432), were obtained from Santa Cruz Biotechnology, Dallas, TX USA.

### 4.1. Cell Culture

The human breast cancer cell lines MCF-7, MDA-MB-231, and MDA-MB-453 were purchased from the National Centre for Cell Science (NCCS), Pune, India. The cells were cultured in DMEM supplemented with 10% FBS and 1% penicillin/streptomycin as antibiotics in a T-25 flask at 37 °C in a humidified atmosphere of 5% CO_2_ in a CO_2_ incubator (Eppendorf, Germany). All experiments were performed using cells from passage 15 or less.

### 4.2. Peptide Synthesis

The chemical structure of the peptide SKACP003 was drawn using ChemSketch software [30]. Using PS-TPGDD (Polystyrene tripropylene glycerolate diacrylate diol) resin as a solid support, SKACP003 was synthesized using the improved Fmoc Solid Phase Peptide Synthesis (SPPS) strategy and then purified with high-performance liquid chromatography (HPLC) [63]. After that, the mass spectrometry (MS) method was used to validate the peptide’s completeness. The amorphous form of the white-colored peptide was kept at −20 °C in an airtight, small glass tube. The use of HPLC allowed the peptide to be determined to be >95% pure.

### 4.3. Cell Cycle Assay

Flow cytometry is one of the most effective tools for analyzing the mode of cell death induced by a tested drug [64]. Breast cancer cell lines MCF-7, MDA-MB-231, and MDA-MB-453 were each cultured with a total of about 5 × 10^5^ cells and exposed to SKACP003 in culture medium at their respective IC_50_ concentrations (193 ± 0.5 µM for MCF-7, 212 ± 0.5 µM for MDA-MB-231, and 156 ± 0.5 µM for MDA-MB-453). The control cells were only provided with the growth medium. The cells were harvested at the end of the 24 h incubation at 37 °C, washed with a PBS solution devoid of calcium and magnesium, fixed with 70% ethanol, and then frozen at 4 °C for 24 h. The cells were washed twice with Dulbecco’s phosphate-buffered saline (DPBS) before being re-suspended in 500 μL of propidium iodide (PI) solution containing 1 μL RNase A (10 μg/μL), 1 μL PI (10 μg/μL), and 0.5% Tween-20 in 500 μL of DPBS and incubated for 30 min at room temperature in the dark before analysis. Cells were collected using an argon-ion laser at 488 nm on a FACS Calibur II system (Becton 52 Dickinson, San Jose, CA), and the cell-cycle distribution was analyzed using the FlowJO software. Typically, 10,000 events were collected for each sample.

### 4.4. Reverse Transcriptase Polymerase Chain Reaction

Total mRNA was extracted using the TRIzol reagent from MCF-7, MDA-MB-231, and MDA-MB-453 breast cancer cell lines under control and treatment conditions (Takara, Japan). The cDNA for each RNA was synthesized using a First Strand cDNA Synthesis Kit and reverse transcriptase-mediated polymerase chain reaction (RT-PCR) with 1 µg of RNA as the template (Takara, Japan). To amplify the cDNA, the Emerald PCR Master Mix (Takara, Japan) was used, with specific primers (Table 1) of the genes of interest used under congruent reaction conditions. The thermal conditions used were as follows: initial denaturation at 94 °C for 2 min, followed by 30–35 cycles of denaturation at 94 °C for 30 s, annealing at appropriate Tm specific for the primers for 1 min, extension at 72 °C for 30 s, with a final extension at 72 °C for 7 min, and holding at 4 °C. β-actin was used as an internal control. The PCR products were resolved in 1% agarose.

### 4.5. Western Blot Analysis

All control and treated breast cancer cell lines were washed several times with warm PBS before being lysed on ice with 200 µL RIPA Lysis Buffer (Santa Cruz Biotechnology, Dallas, TX, USA), proteinase inhibition, and a phosphatase inhibitor cocktail (Santa Cruz Biotechnology, Dallas, TX, USA). Following a 20-min incubation, lysates were cleared by centrifugation at 10,000 rpm for 15 min at 4 °C. Supernatants were quantified using Lowry’s method, and absorbance at 655 nm was measured using a microplate reader.

Protein samples extracted from both the treated and control cells (50 μg) were added to a 4X sodium dodecyl sulfate-polyacrylamide gel electrophoresis (SDS PAGE) along with 20% glycerol, and sample buffer (100 mmol/L Tris-HCl, 4% SDS, 0.2% bromophenol blue, and 5% mercaptoethanol). The samples were boiled for 10 min at 95 °C before being resolved on 12% SDS-PAGE at 100 V for 2 h. Then, protein samples were transferred into nitrocellulose membranes in a transfer buffer containing 39 mmol/L glycine, 20% methanol, and 48 mmol/L Tris for 120 min at 220 mAmp. Following this, the membranes were blocked for 2 h in a solution of 5% skim milk diluted in Tris-buffered saline (TBS) containing 0.1% Tween 20 (TBST). Next, the membranes were incubated overnight at 4 °C with a specific primary antibody (1:1000 dilution, SIGMA, Ronkokoma, NY, USA). β-catenin and β-actin primary antibodies were used to determine β-catenin and β-actin protein levels. Following incubation with the secondary antibodies (goat anti-mouse Ig-ALP antibody; Santa Cruz Biotechnology, Dallas, TX, USA, 1:1000 dilution) for 2 h at 4 °C, the blots were washed three times for 10 min in TBST. The blots were then washed three times in TBS for 5 min, three times in TBST for 10 min, and developed with BCIP/NBT (SIGMA, Ronkokoma, NY, USA).

### 4.6. Statistical Analysis

A two-way ANOVA with Dunnett’s post-test was performed using Graphpad Prism, version 6.04 (La Jolla, CA, USA), to compare treated cell lines with the controls. A *p* < 0.05 value was considered statistically significant. All the results were expressed as mean value ± standard deviation.

## 5. Conclusions

SKACP003 is effective in all measures of cancer elimination activity. Therefore, it may be integral to developing a more potent and side-effect-free drug for breast cancer treatment. Along these lines, additional research, such as clinical trials, might be continued in the future. Furthermore, to achieve the highest possible levels of selectivity, feasibility, and safety, SKACP003 may be combined with chemotherapy or nanotechnology.

## Figures and Tables

**Table 1:** List of forward and reverse primers used in this study.

SN	Gene	Forward Primer	Reverse Primer	Annealing Temperature (°C)
1.	*C-Myc*	5′-TGCCTTGGTTCATCTGGGTC-3′	5′-GCTTAGGAGTGCTTGGGACA-3′	53.8
2.	*BCL2*	5′-CTTTGAGTTCGGTGGGGTCA-3′	5′-GGGCCGTACAGTTCCACAAA-3′	53.8
3.	*BAX*	5′-CGTGTCTGATCAATCCCCGAT-3′	5′-AGCTAGGGTCAGAGGGTCAT-3′	54.4
4.	*CDK4*	5′-ACCAGATGGCACTTACACCC-3′	5′-GGCAGCCCAATCAGGTCAAA-3′	53.8
5.	*CDK6*	5′-ACAGAGCACCCGAAGTCTTG-3′	5′-GGGAGTCCAATCACGTCCAA-3′	53.8
6.	*MMP 2*	5′-TGATGGCATCGCTCAGATCC-3′	5′-GGCCTCGTATACCGCATCAA-3′	53.8
7.	*MMP 7*	5′-CAATTGTCTCTGGACGGCAG-3′	5′-CTGAGCCTGTTCCCACTGTA-3′	53.8
8.	*CASP9*	5′-CATCCCAGGAAGGCAACAAG-3′	5′-GGGAAGCATGGCTAGGACTC-3′	53.8
9.	*CASP8*	5′-GGCTTTGACCACGACCTTTG-3′	5′-TCAGTGCCATAGATGATGCCC-3′	54.1
10.	*CASP3*	5′-GCAAGTTACAGTGATGCTGTGC-3′	5′-CCATGCCCACAGATGCCTAA-3′	54.3
11.	*COX2*	5′-GTCTGGTGCCTGGTCTGATG-3′	5′-GCCACTCAAGTGTTGCACAT-3′	53.9
12.	*P* *^68^*	5′-GGGATGGCCAGTTGCTCTAA-3′	5′-AGCACCAAACAAATAGGCCC-3′	52.8
13.	*VEGF-A*	5′-GTATAAGTCCTGGAGCGTTCCCT-3′	5′-TTTAACTCAAGCTGCCTCGCC-3′	61.0

## Data Availability

All data used in this study have been included in the manuscript.

## Sample Availability

Samples of SKACP003 are available from the authors.
